# Community-Based Values for 2009 Pandemic Influenza A H1N1 Illnesses and Vaccination-Related Adverse Events

**DOI:** 10.1371/journal.pone.0027777

**Published:** 2011-12-19

**Authors:** Tara A. Lavelle, Martin I. Meltzer, Achamyeleh Gebremariam, Kara Lamarand, Anthony E. Fiore, Lisa A. Prosser

**Affiliations:** 1 Ph.D. Program in Health Policy, Harvard University, Cambridge, Massachusetts, United States of America; 2 The Center for Health Decision Science, Harvard School of Public Health, Boston, Massachusetts, United States of America; 3 Influenza Division, National Center for Immunization and Respiratory Diseases, Centers for Disease Control and Prevention, Atlanta, Georgia, United States of America; 4 Child Health Evaluation and Research Unit, Division of General Pediatrics, University of Michigan Health System, Ann Arbor, Michigan, United States of America; INSERM and Université Pierre et Marie Curie, France

## Abstract

**Objective:**

To evaluate community-based values for avoiding pandemic influenza (A) H1N1 (pH1N1) illness and vaccination-related adverse events in adults and children.

**Methods:**

Adult community members were randomly selected from a nationally representative research panel to complete an internet survey (response rate = 65%; n = 718). Respondents answered a series of time trade-off questions to value four hypothetical health state scenarios for varying ages (1, 8, 35, or 70 years): uncomplicated pH1N1 illness, pH1N1 illness-related hospitalization, severe allergic reaction to the pH1N1 vaccine, and Guillain-Barré syndrome. We calculated descriptive statistics for time trade-off amounts and derived quality adjusted life year losses for these events. Multivariate regression analyses evaluated the effect of scenario age, as well as respondent socio-demographic and health characteristics on time trade-off amounts.

**Results:**

Respondents were willing to trade more time to avoid the more severe outcomes, hospitalization and Guillain-Barré syndrome. In our adjusted and unadjusted analyses, age of the patient in the scenario was significantly associated with time trade-off amounts (p-value<0.05), with respondents willing to trade more time to prevent outcomes in children versus adults. Persons who had received the pH1N1 vaccination were willing to trade significantly more time to avoid hospitalization, severe allergic reaction, and Guillain-Barré syndrome, controlling for other variables in adjusted analyses.(p-value<0.05)

**Conclusions:**

Community members placed the highest value on preventing outcomes in children, compared with adults, and the time trade-off values reported were consistent with the severity of the outcomes presented. Considering these public values along with other decision-making factors may help policy makers improve the allocation of pandemic vaccine resources.

## Introduction

In April 2009, the first influenza pandemic in over forty years began in North America; the causative virus was 2009 pandemic influenza (A) H1N1 (pH1N1). Under guidance from the Advisory Committee for Immunization Practices (ACIP), the Centers for Disease Control and Prevention recommended target groups for vaccination [1]. A vaccine became available during October 2009, and a program was implemented on an emergency basis to reduce the impact of the expanding pandemic.

Vaccination programs, such as the one implemented for pH1N1, involve an inherent trade-off of risks. Vaccinating for a particular disease reduces the risk of infectious illness, but introduces new risks of vaccine-related adverse events. The acceptability of a vaccination program depends in part on how the public values the potential risks and benefits of vaccination. By examining the likelihood of these risks and benefits, as well the value of prevention, decision makers can determine the potential value of a public vaccination program. When pH1N1 vaccine recommendations were made in the U.S. the only studies reporting community values associated with influenza illness and vaccination were based on data from seasonal influenza [2], [3], [4]. Outcomes related to pH1N1 illness and vaccination may be valued differently, however. We present in this study estimates of community-based values for avoiding adult and pediatric health events related to pH1N1 illness and vaccination.

## Methods

### Ethics Statement

This study was reviewed and provided with exempt status by the University of Michigan institutional review board. All study data were de-identified; no informed consent was required by the board in order for individuals to participate in the study.

### Overview

We used the time trade off (TTO) approach to evaluate community-based values for avoiding pH1N1 illness and vaccination-related adverse events in adults and children. The TTO method estimates the value each respondent puts on avoiding a particular health outcome by estimating their willingness to trade quantity of life for quality of life. For example, a TTO question may value diabetes prevention by measuring the amount of time a person would be willing to give up from her life span to avoid living with diabetes (living instead a reduced number of years without diabetes). The resulting TTO values can be interpreted as subjective measures of quality of life, and are the basis for constructing quality adjusted life years (QALYs). QALYs are created by weighting a segment of time spent in a specific health state by the quality of life value associated with that health state. QALYs have been used to measure the morbidity associated with chronic illness over an extended time period [5]. In our study, to value the morbidity associated with the health states of pH1N1 illness and vaccination-related adverse events, we used TTO responses from our survey to calculate short-term QALY losses.

### Study participants

We randomly sampled adult community members to complete an internet survey from a research panel designed to be statistically representative of the U.S. general adult population. The survey was administered by Knowledge Networks (Menlo Park, CA), which currently recruits new research panel members by mail from a published address-based sample frame that covers approximately 98% of U.S. households [6]. Non-internet households who choose to join the panel are provided with internet access and a laptop computer. Households who use their own computer and internet service to answer online surveys administered by Knowledge Networks receive small monthly stipends in exchange for their participation [7]. Demographic information collected for all new panel members includes gender, age, ages of their household members, race/ethnicity, income, and education level.

### Study Procedures

Participation in the study required completion of a 15-minute survey during January 2010. Respondents answered a series of TTO questions to value hypothetical health state scenarios describing: uncomplicated pH1N1 illness, pH1N1 illness-related hospitalization, severe allergic reaction to the pH1N1 vaccine, and Guillain-Barré syndrome, a potential vaccine-related adverse event. Each of the 4 health state scenarios had 4 versions; each referencing a hypothetical person aged 1, 8, 35, and 70 years. Respondents were randomly assigned to value 2 different ages for each of the 4 scenarios, for a total of 8 TTO questions. The different age versions of each health state scenario were identical except for the description of usual activities, which included school/daycare for children and work/household responsibilities for adults. We instructed respondents to imagine a family member or friend that closely matched the age description in the scenario at hand. Respondents were also asked whether they had been vaccinated for pH1N1 or seasonal influenza, and whether they or anyone else in their family had ever experienced pH1N1 or seasonal influenza illness or an influenza vaccination-related adverse event.

### TTO estimation

**Figure 1:**
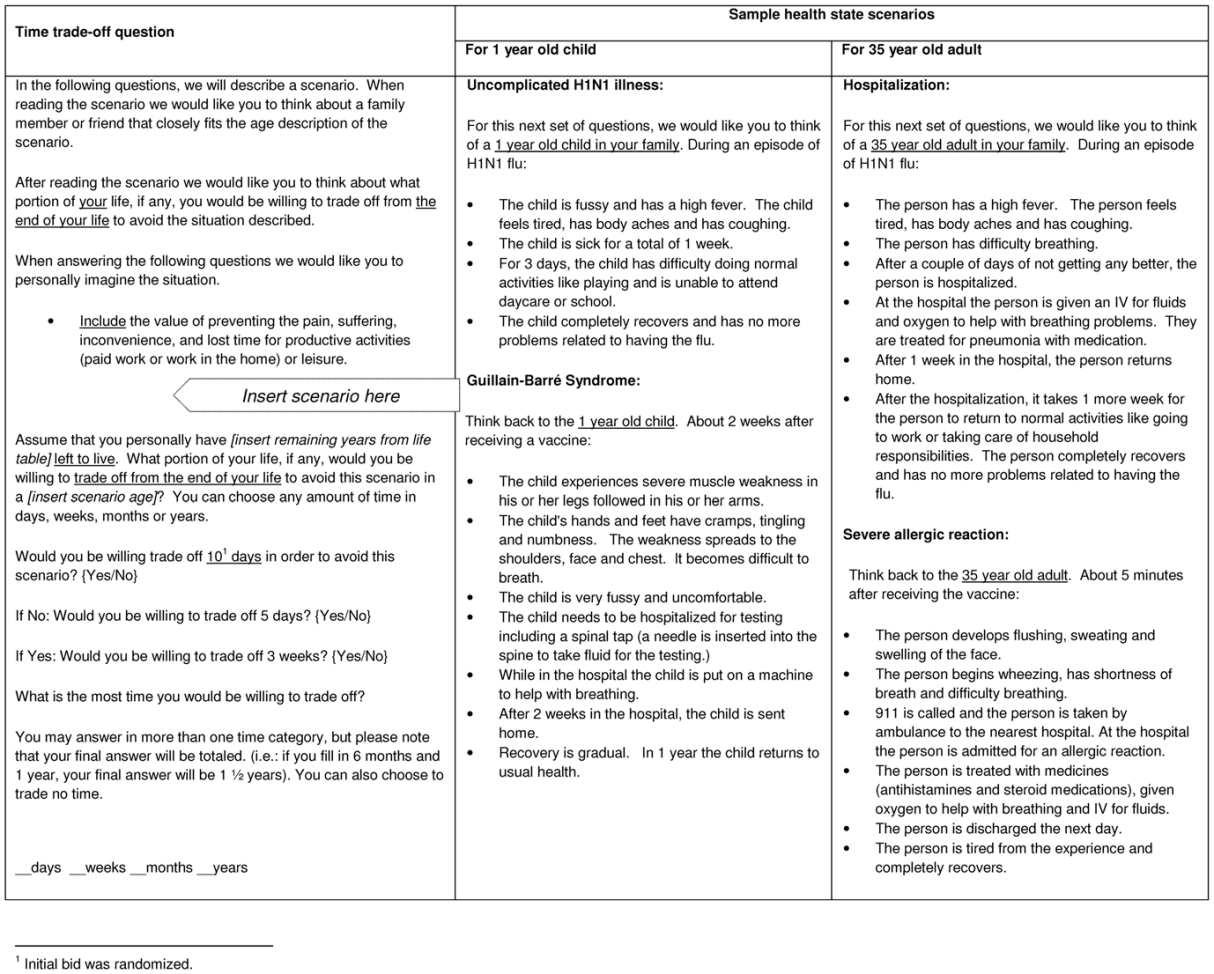
Time trade off question with sample health scenarios.

We used a modified bidding algorithm, combining binary and open ended response questions, to measure TTO amounts. This method is less prone to non-response problems compared to a single open ended question [8]. After presenting one age-specific health event related to pH1N1 illness or vaccination, we first asked respondents whether they would trade a fixed amount of time from the end of their life in exchange for avoiding the health event. (Figure 1) The amount of time that the respondents were asked to trade was randomized to reduce anchoring bias, with initial TTO amounts ranging from 2 days to 2 months for uncomplicated flu and severe allergic reaction outcomes, and 2 weeks to 1 year for hospitalization and Guillain-Barré syndrome outcomes. A follow up binary question offered a higher TTO amount if the initial response was “yes,” and a lower TTO amount if the initial response was “no.” These two binary questions were followed by an open-ended question which asked respondents for the maximum amount of time they would trade from the end of their life (in days, weeks, months, and years) to avoid the health state in question; this maximum TTO value was used for all analyses.

### Analyses

We calculated descriptive statistics for TTO data, including means, medians, 5^th^ and 95^th^ percentiles, minimums and maximums. Confidence intervals around mean values were estimated using bootstrapping with replacement procedures [9]. We used the Kruskal-Wallis non-parametric test in unadjusted analyses to evaluate whether median values differed by scenario age. All summary statistics used unweighted data, due to the similarity between unweighted and weighted summary statistics. In our primary analysis, TTO amounts greater than life expectancy were reset to equal the respondent's life expectancy, and we evaluated the effect of this in sensitivity analyses.

As respondents were asked their willingness to trade time from the end of their life, we adjusted for the potential impact of time preference by using a 3% discount rate to calculate discounted TTO values [5], [10]. Dividing the respondents' discounted TTO amount by their discounted life expectancy allowed us to calculate a short term QALY loss associated with the temporary health state in question.

To evaluate the association between TTO amounts and respondent/scenario characteristics, we used a generalized estimating equation negative binomial regression model. This type of regression model is bounded at 0 to account for the lower limit of TTO responses and adjusts for the correlations associated with multiple evaluations per respondent [11]. Using the undiscounted TTO amounts reported for the four different health states as the dependent variables, the four final regression models each included as independent variables: scenario age, gender, respondent age, education, race/ethnicity, having a child under the age of 18, vaccination status, and experience with the health state in question. The goodness of fit of each model was measured using a test of concordance between the observed and predicted TTO values [12].

## Results

### Respondents

The survey was sent to 1,110 members of the survey panel. Of those invited by email to participate in the online survey, 65% completed the survey (n = 718); 9% of respondents were eliminated from the primary analysis due to missing or invalid responses, leaving a final analysis sample size of n = 659. Observations were excluded from the analysis if TTO amounts were missing for more than half (4 or more) of the scenarios (n = 56), the responses in all four time metrics were equivalent (n = 2), or the TTO amount was nonsensical (e.g., 999999 months) (n = 1).

Demographic characteristics among those who responded to the survey were statistically different from those who did not respond to the survey for all demographic characteristics except location (country region and metropolitan status). Compared to non-responders, responders were more likely to be male, white, married, aged 45 or older, college educated, and earn more than $35,000 annually; respondents were also less likely to have a child under the age of 18 years. (p<0.05 for all)

Respondent characteristics included in the primary analysis are summarized in Tables 1, and 2. Without survey weights, 50% of respondents were male, 56% were married, 78% were white, non-Hispanic, 33% had a child under the age of 18 living at home, and 84% rated themselves in excellent/very good or good overall health. Forty two percent of all respondents had received the seasonal flu vaccine in the previous 12 months, and 21% had received the pH1N1 vaccine in this time period. Thirty one percent of respondents reported that they had experienced seasonal influenza themselves, and 18% had a family member who had experienced this illness at some point in the past. Three percent of respondents had experienced pH1N1 illness themselves, and 5% had a family member who had experienced this illness. Only a small fraction of respondents (<1–2%, depending on question) reported that they had experienced a hospitalization related to influenza, or a side effect from an influenza vaccine, either personally or through a family member.

**Table 1:** Respondent demographic characteristics (n = 659).

	Frequency	
Characteristic	Unweighted	Weighted^1^
Gender		
Male	49.9%	49.8%
Female	50.1%	50.2%
Age		
18–29	15.9%	20.6%
30–44	24.4%	27.7%
45–59	33.9%	27.7%
60+	25.8%	24.0%
Education		
Less than High School	11.2%	12.4%
High School	33.6%	30.3%
Some College	26.4%	28.3%
Bachelor's degree or higher	28.8%	29.0%
Race		
White, Non-Hispanic	77.7%	70.4%
Black, Non-Hispanic	7.7%	10.4%
Other, Non-Hispanic	2.6%	5.4%
Hispanic	10.2%	12.7%
2+ races, Non-Hispanic	1.8%	1.1%
Marital Status		
Married	55.5%	52.3%
Single (never married)	21.5%	23.3%
Divorced	11.1%	12.8%
Widowed	6.1%	5.0%
Separated	1.2%	2.0%
Living with partner	4.6%	4.6%
Household Income		
<3 times poverty level	44.3%	43.2%
≥3 times poverty level	45.4%	45.3%
Don't Know	10.3%	11.5%
Regions		
Northeast	19.3%	19.1%
Midwest	21.5%	20.4%
South	36.1%	37.8%
West	23.1%	22.7%
Global Health		
Excellent/Very Good	47.6%	45.8%
Good	36.0%	38.1%
Fair	13.5%	13.4%
Poor	2.9%	2.7%
Child Under 18 Living at Home	32.5%	32.5%
TTO questions hard to answer	51.0%	51.4%
Households with Internet	62.2%	63.6%

1 Post stratification weights were provided by Knowledge Networks to account for sampling and non-response bias.

**Table 2:** Respondent influenza-related characteristics (n = 659).

	Frequency	
Characteristic	Unweighted	Weighted^1^
Received pH1N1 Vaccine in past 12 months	20.8%	19.8%
Received Seasonal Influenza Vaccine in past 12 months	41.7%	40.2%
Influenza Illness, Summary		
Experienced pH1N1 Influenza Illness, Self	3.2%	4.3%
Experienced pH1N1 Influenza Illness, Family Member	5.3%	5.3%
Experienced Seasonal Influenza Illness, Self	31.2%	32.3%
Experienced Seasonal Influenza Illness, Family Member	17.5%	17.7%
Influenza-Related Hospitalization, Summary		
Experienced pH1N1-Related Hospitalization, Self	0.2%	0.1%
Experienced pH1N1-Related Hospitalization, Family member	1.8%	1.8%
Experienced Seasonal Influenza-Related Hospitalization, Self	0.8%	1.3%
Experienced Seasonal Influenza-Related Hospitalization, Family Member	2.6%	3.0%
Vaccine-related Severe Allergic Reaction, Summary		
Experienced Severe Allergic Reaction to pH1N1 vaccine, Self	0.5%	0.5%
Experienced Severe Allergic Reaction to pH1N1 vaccine, Family Member	0.2%	0.1%
Experienced Severe Allergic Reaction to Seasonal Influenza vaccine, Self	1.4%	1.5%
Experienced Severe Allergic Reaction to Seasonal Influenza vaccine, Family Member	1.2%	1.2%
Vaccine-related Guillain-Barré Syndrome, Summary		
Experienced pH1N1 Guillain-Barré Syndrome, Self	0.3%	0.4%
Experienced pH1N1 Guillain-Barré Syndrome, Family Member	0.0%	0.0%
Experienced Seasonal Influenza Guillain-Barré Syndrome, Self	0.2%	0.2%
Experienced Seasonal Influenza Guillain-Barré Syndrome, Family Member	0.3%	0.6%

1 Post stratification weights were provided by Knowledge Networks to account for sampling and non-response bias.

### Descriptive statistics

Respondents were willing to trade a median of 7 undiscounted days to avoid a hospitalization related to pH1N1 influenza and 30 days to avoid Guillain-Barré syndrome, compared to a median of 2 and 4 undiscounted days to avoid uncomplicated pH1N1 illness and severe allergic reaction, respectively (Table 3). Due to the right skewed distribution for TTO amounts in all 4 health states (unsymmetrical, with the greatest proportion of respondents willing to trade 0 days), mean values were substantially higher and more variable than median values. Respondents were willing to trade a mean of 291 and 376 undiscounted days to avoid a hospitalization and Guillain-Barré syndrome, and a mean of 226 and 222 undiscounted days to avoid uncomplicated pH1N1 illness and a severe allergic reaction, respectively. (Table 3)

**Table 3:** Time-tradeoff amounts for 2009 pandemic influenza (A) H1N1 illness and vaccination-related adverse events^1^.

		Undiscounted time-tradeoff amounts (days from end of life)							Discounted (3%) time-tradeoff amounts (days)						
						Percentile		Range					Percentile		Range
Health Scenario	n	Mean	95% CI^2^	Median	p-value^3^	5th	95th	min – max	Mean	95% CI^2^	Median	p-value^3^	5th	95th	min – max
Uncomplicated pH1N1 illness															
1-year old	296	415	268–668	3		0	1826	0 – 13514	242.2	145–406	1.1		0	870.6	0 – 8217
8-year old	345	261	170–432	3		0	1315	0 – 13514	137.0	81–242	1.2		0	458.1	0 – 8217
35-year old	349	155	103–244	1		0	731	0 – 6940	75.53	48–135	0.30		0	391.8	0 – 5310
70-year old	307	85	59–126	2		0	470	0 – 2726	43.20	29–66	0.72		0	252.4	0 – 1508
All ages	1297	226	177–293	2	0.0177	0	731	0 – 13514	122.3	91–164	0.66	0.0436	0	440.1	0 – 8217
pH1N1 illness-related hospitalization															
1-year old	297	442	303–682	14		0	1826	0 – 13514	242.0	153–394	5.1		0	940.0	0 – 8217
8-year old	342	374	258–558	14		0	1826	0 – 13514	190.4	129–316	4.5		0	877.7	0 – 8217
35-year old	358	242	159–400	7		0	731	0 – 10958	125.2	80–222	1.9		0	497.5	0 – 6906
70-year old	307	108	79–157	7		0	444	0 – 3653	52.04	36–77	2.6		0	245.1	0 – 1650
All ages	1304	291	235–362	7	0.0003	0	1391	0 – 13514	151.7	118–197	3.2	0.0007	0	527.8	0 – 8217
Severe allergic reaction															
1-year old	291	313	196–516	7		0	911	0 – 13514	183.3	108–330	2.9		0	392.7	0 – 8217
8-year old	341	307	208–499	6		0	1695	0 – 13514	156.0	98–259	2.1		0	608.5	0 – 8217
35-year old	352	180	112–321	2		0	545	0 – 10958	94.02	54–189	0.50		0	251.5	0 – 7266
70-year old	301	88	64–141	2		0	500	0 – 3653	40.03	28–62	0.82		0	245.1	0 – 1650
All ages	1285	222	176–289	4	0.0002	0	731	0 – 13514	118.0	90–160	1.2	0.0147	0	337.3	0 – 8217
Guillain-Barré Syndrome															
1-year old	292	527	391–774	60		0	2192	0 – 13514	279.3	189–421	26.7		0	941.1	0 – 8217
8-year old	340	488	362–685	60		0	2009	0 – 13514	243.2	170–373	22.0		0	960.5	0 – 8217
35-year old	351	330	229–479	30		0	1096	0 – 10958	173.2	118–276	8.4		0	543.6	0 – 7266
70-year old	303	158	122–244	28		0	545	0 – 6544	73.45	56–114	8.0		0	283.7	0 – 3351
All ages	1286	376	317–454	30	0.0001	0	1641	0 – 13514	192.3	157–244	13.6	0.0001	0	614.9	0 – 8217

1 Using unweighted data.

2 To generate confidence intervals around our mean values, we used bootstrap re-sampling of size equal to the sample size (approximately 1300, depending on the health state) with 3000 iterations. From each of the 3000 bootstrap samples generated, we calculate the overall means and means by scenario age to create a sampling distribution around the original mean values.

3 Kruskal-Wallis test evaluated whether median values differed by scenario age.

When stratified by scenario age within each health state, median TTO amounts differed significantly by age (p-value<0.05 for all health states). (Table 3) On average, respondents were willing to trade more time to avoid pH1N1-related illnesses and vaccination-related adverse events in children, compared to adults. Respondents were willing to trade a median of 3 and 14 undiscounted days to avoid pH1N1 illness and hospitalization in a 1 year old child, but were only willing to trade a median of 2 and 7 days to avoid these same outcomes in a 70 year old adult. Likewise, respondents were willing to trade a median of 7 and 60 undiscounted days to prevent a severe vaccine allergic reaction and Guillain-Barré syndrome in a 1 year old child, but were only willing to trade a median of 2 and 28 days, to avoid these same outcomes in a 70 year old adult. (Table 3)

The median values for the loss in QALYs from pH1N1 illness and vaccination-related adverse events also exhibited a significant difference by scenario age (p-value<0.05 for all health states). (Table 4) For example, pH1N1-related hospitalization was associated with a 0.0007 median QALY loss for a 1 year old and a 0.0003 median QALY loss for a 70 year old. Likewise, Guillain-Barré syndrome was associated with a 0.0039 median QALY loss for a 1 year old and a 0.0012 median QALY loss for a 70 year old. Mean values were consistently higher and more variable than median values. (Table 4)

**Table 4:** Loss in quality adjusted life years (QALYs) for 2009 pandemic influenza (A) H1N1 illness and vaccination-related adverse events^1^.

			95% CI^2^				Percentile		Range
Loss in QALYs	n	Mean	Lower bound	Upper bound	Median	p-value^3^	5^th^	95^th^	min – max
Uncomplicated pH1N1 illness									
1-year old	296	0.0394	0.0251	0.0625	0.0001		0	0.1708	0 – 1.0000
8-year old	345	0.0218	0.0132	0.0377	0.0001		0	0.0907	0 – 1.0000
35-year old	349	0.0138	0.0085	0.0247	0.0000		0	0.0551	0 – 1.0000
70-year old	307	0.0092	0.0059	0.0159	0.0001		0	0.0427	0 – 0.5226
All ages	1297	0.0207	0.0156	0.0267	0.0001	0.0211	0	0.0802	0 – 1.0000
pH1N1 illness-related hospitalization									
1-year old	297	0.0391	0.0266	0.0613	0.0007		0	0.1851	0 – 1.0000
8-year old	342	0.0304	0.0200	0.0477	0.0006		0	0.1174	0 – 1.0000
35-year old	358	0.0217	0.0140	0.0362	0.0003		0	0.1125	0 – 1.0000
70-year old	307	0.0104	0.0073	0.0160	0.0003		0	0.0496	0 – 0.4034
All ages	1304	0.0253	0.0202	0.0320	0.0004	0.0005	0	0.1118	0 – 1.0000
Severe allergic reaction									
1-year old	291	0.0317	0.0195	0.0536	0.0003		0	0.0853	0 – 1.0000
8-year old	341	0.0251	0.0162	0.0401	0.0003		0	0.1017	0 – 1.0000
35-year old	352	0.0166	0.0092	0.0325	0.0001		0	0.0496	0 – 1.0000
70-year old	301	0.0074	0.0051	0.0114	0.0001		0	0.0366	0 – 0.2183
All ages	1285	0.0201	0.0156	0.0266	0.0002	0.0002	0	0.0691	0 – 1.0000
Guillain-Barré Syndrome									
1-year old	292	0.0475	0.0329	0.0692	0.0039		0	0.1533	0 – 1.0000
8-year old	340	0.0391	0.0281	0.0584	0.0034		0	0.1381	0 – 1.0000
35-year old	351	0.0300	0.0204	0.0460	0.0012		0	0.1300	0 – 1.0000
70-year old	303	0.0135	0.0103	0.0194	0.0012		0	0.0640	0 – 0.4433
All ages	1286	0.0325	0.0268	0.0403	0.0019	0.0001	0	0.1236	0 – 1.0000

1 Using unweighted data.

2 To generate confidence intervals around our mean values, we used bootstrap re-sampling of size equal to the sample size (approximately 1300, depending on the health state) with 3000 iterations. From each of the 3000 bootstrap samples generated, we calculate the overall means and means by scenario age to create a sampling distribution around the original mean values.

3 Kruskal-Wallis test evaluated whether median values differed by scenario age.

### Regression analyses

After adjusting for respondent characteristics, the 1 year and 8 year old scenario ages were significantly associated with greater TTO amounts (compared with the 35 year old scenario age) in all four final regression models. (p-values<0.05, Table 5) Seventy year old scenario age was significantly associated with lower TTO amounts (compared to the 35 year scenario age) in the final regression models for hospitalization and Guillain-Barré syndrome outcomes.

**Table 5:** Multivariate regression results: Time trade-off amounts by scenario age, sociodemographics, illness experience, and vaccination status, predicted number of days traded.

	Uncomplicated pH1N1 Illness^1^		pH1N1 Illness-related Hospitalization^1^		Severe Allergic Reaction^1^		Guillain-Barré syndrome^1^	
Predictors	Estimate	SE	Estimate	SE	Estimate	SE	Estimate	SE
**Scenario Age**								
1 year	212.06*	37.01	315.06*	58.43	179.30*	27.74	362.55*	37.06
8 years	133.87*	22.99	251.47*	38.29	178.54*	29.05	333.34*	31.00
35 years#	88.78	14.85	181.07	30.35	102.06	16.23	242.78	25.73
70 years	82.08	14.85	122.77*	20.45	80.64	13.96	189.30*	18.70
**Gender**								
Male	134.10	26.92	200.55	41.21	108.78	20.23	229.98	29.66
Female#	104.70	17.07	208.23	32.77	148.69	27.87	322.87	41.48
**Respondent Age**								
18–29 yrs#	42.86	12.81	122.78	47.80	77.00	20.49	164.66	31.36
30–44 yrs	127.30*	43.66	262.50	85.75	123.10	44.75	321.42*	76.84
45–59 yrs	119.70*	29.61	215.97	52.96	143.05	35.93	277.05*	47.67
60 & above	207.41*	51.82	205.09	44.66	153.84	40.95	312.47*	54.33
**Education**								
<High School	307.60*	112.78	271.93*	82.06	197.52*	55.70	328.23*	68.15
High School	231.97*	55.77	322.30*	69.34	295.44*	77.68	512.44*	97.07
Some College	159.83*	34.98	266.07*	75.05	125.63*	30.76	239.68*	44.32
Bachelors & above#	37.24	10.28	84.92	22.89	40.57	10.34	139.32	19.30
**Race/Ethnicity**								
White, Non- Hispanic#	100.84	16.67	179.64	28.27	102.87	16.67	248.76	25.90
Black, Non- Hispanic	538.04*	192.98	456.50	206.98	299.25*	87.53	406.62	125.60
Other, Non- Hispanic	55.84	27.92	143.50	65.01	113.87	39.06	246.96	133.04
Hispanic	383.92*	109.84	342.61	125.27	342.63*	117.27	421.06	132.49
**Children <18 yrs**								
No#	144.92	29.99	216.02	42.25	132.97	26.97	244.80	32.11
Yes	98.31	21.74	182.12	44.81	116.23	24.89	340.97	62.31
**Experience with Illness (self/family)**								
No#	78.28	12.19	201.51	26.82	125.72	17.08	271.10	24.55
Yes	236.97*	54.85	298.85	144.91	221.94	106.39	1234.26*	688.48
**pH1N1 Vaccination Status**								
No#	115.07	17.92	177.67	27.00	108.86	16.52	242.64	24.51
Yes	189.87	46.87	347.31*	90.24	231.03*	64.04	425.03*	92.75

*p-value<0.05; indicates statistical difference of value compared to the reference group.

# Reference group.

1 Model goodness-of-fit concordance coefficients and confidence intervals- uncomplicated pH1N1 illness: 0.129 (95% CI:0.090, 0.168), pH1N1 illness-related hospitalization: 0.071 (95% CI: 0.051,0.092), Severe Allergic Reaction: 0.095 (95% CI: 0.071,0.118), Guillain-Barré syndrome: 0.112 (95% CI: 0.089,0.135).

For all four health states, having less than a college degree was significantly associated with greater TTO amounts. (Table 5) Other demographic characteristic associations were not consistent across outcomes, however. Compared with a white, non-Hispanic reference group, being Hispanic or black, non-Hispanic, was significantly associated with greater TTO amounts for uncomplicated pH1N1 illness and allergic reaction only. Being over the age of 30 was significantly associated with greater TTO amounts for uncomplicated pH1N1 illness and Guillain-Barré syndrome only.

Respondent health characteristic associations were also inconsistent predictors of TTO amounts. Experience with uncomplicated pH1N1 illness and Guillain-Barré syndrome was significantly associated with greater TTO amounts for those respective health states, but experience with pH1N1-related hospitalization and severe allergic reaction was not significantly associated with the TTO amounts for these outcomes. Compared to respondents that had not been vaccinated for pH1N1, those that had been vaccinated were willing to trade significantly more time to avoid a pH1N1-related hospitalization (p-value = 0.03) but were also willing to trade more time to avoid both vaccination related adverse events. (p-value<0.05 for both)

Respondent's gender, and having a child under 18, did not significantly impact TTO responses. Concordance coefficients, used to measure the goodness of fit of our models, ranged from 0.071 to 0.129. All coefficients were significantly greater than zero, indicating that there was a significant and positive correlation between our observed and predicted TTO values. (Table 5) Sensitivity analyses which excluded respondents who traded amounts larger than life expectancy yielded very similar results to the primary analysis, which included these respondents with their TTO amounts reset to their life expectancy (results not shown).

## Discussion

This study reports community values for avoiding pH1N1 illness-related outcomes and vaccination-related adverse events in the U.S. On average, respondents' values for avoiding pH1N1-related health events and vaccination-related adverse events were aligned with the portrayed severity of these events in our survey. Compared to pH1N1 illness-related hospitalization, respondents were willing to trade less time to avoid uncomplicated pH1N1 illness and a severe allergic reaction from vaccination, across all scenario ages. Respondents were willing to trade the greatest amount of time to avoid the most severe outcome, Guillain-Barré syndrome. This relative ranking of these TTO values across outcomes is consistent with previous findings for outcomes associated with seasonal influenza illness and vaccine related adverse events [3]. In regression analyses, 1 year and 8 year old scenario ages were consistently associated with greater TTO amounts, indicating that the public may give preference to preventing pH1N1 illness and vaccine- related health outcomes in children compared with adults. These data are consistent with earlier findings that indicate that community members may prefer to prioritize child health [3], [13], [14].

These findings are also consistent with the ACIP's recommendations in July of 2009 which stated that children and young adults aged 6 months–25 years should be among those prioritized for pH1N1 vaccination, and that children 6 month–4 years should be one of the groups prioritized under a scenario of limited vaccine supply [1]. These recommendations were made based on data of disease prevalence and risk of complications, and some limited data from community engagement exercises performed as part of pandemic preparedness [15]. Also considering these new preference data obtained from community members after the recent pH1N1 influenza pandemic may help policy makers better define key target groups to prioritize for vaccination during the next influenza pandemic.

Our analysis also indicates that certain characteristics of community members may be significant predictors of health state valuations. In adjusted analyses, we found that respondents with less than a bachelor's degree were willing to trade significantly more time than those with a higher level of education to avoid all four health states, controlling for other variables in a multivariate regression. This finding is not consistent with values elicited for seasonal influenza, and may represent a finding that is important to note in light of the novel nature of pH1N1 compared to seasonal influenza [3]. Hispanic and black, non-Hispanic respondents were also willing to trade significantly more time than white respondents to avoid uncomplicated pH1N1 illness and severe allergic reaction. This statistical association between respondent race and health state valuation is consistent with values elicited from community members for seasonal influenza and other health states [3], [16]. Although no consensus exists regarding the cause of the association, one possible explanation is that respondents without a usual source of care may demonstrate a stronger preference to avoid illness. Previous research has shown that compared to white individuals, Hispanic and black individuals are less likely to have a doctor's office as their usual source of care, regardless of insurance coverage, family income and geographic region [17], [18]. As we did not measure usual source of care, it is possible that this variable confounded the race association found in our analysis. Future research should assess respondents' usual source of care and parse out its contribution, along with race and other factors, to health state preferences.

An important limitation of this study is that we used a stated preference approach to value health states. These stated preferences may not reflect the actual choices that these respondents may make when faced with a choice between accepting or rejecting vaccination. In addition, we used the TTO approach for valuing health states, but other methods may have produced different results [19]. As with most vignettes used to estimate preferences, the scenarios used in our survey were concise descriptions of complex health events; adding additional dimensions of health to these vignettes may have influenced respondents' valuations [20].

We also do not know the generalizability of these results. Our measurement of public values for health states related only to this influenza pandemic, and may not relate to more severe influenza pandemics. Another limitation is that both the timing of our survey and the representativeness of the sample may not have been optimal for determining truly representative public values. The survey was fielded after the fall epidemic had passed and the vaccination program had been initiated, and so may not reflect the important public values that were relevant during the time that vaccination program decisions were being made. Data have shown that the public's concern about getting sick from pH1N1 as well as their concern about the safety risks associated with vaccination declined over the duration of the epidemic [21]. Also, compared to non-respondents, our respondents were more likely to be college educated, married, white, older males, and thus may have reported values different from a more population representative sample.

In this study we measured values for health outcomes related to pH1N1 illness and vaccination from the general U.S. public, and not specifically from those that have experienced pH1N1 illness. Previous studies have found that compared to a sample of persons who have not experienced an ill health state, those who have experienced it are typically willing to trade less time to avoid the illness [22], [23], [24]. Many of these studies, however, have focused on chronic illnesses, and there is limited evidence as to how experience or familiarity with a short term health state may influence preferences for avoiding these health outcomes. Van Hoek, et al. estimated a 0.008 QALY loss attributable to pH1N1 in a sample of confirmed pH1N1 cases using the EQ-5D questionnaire. This QALY loss among those who have experienced pH1N1 illness is difficult to compare to our results, however, because it averages over a sample of confirmed cases with and without complications [25]. In our adjusted analyses, we found that those who experienced uncomplicated pH1N1 illness or Guillain-Barré syndrome were willing to trade significantly more time to avoid these health states compared with those without experience.(p-value<0.05 for both) More research is needed to determine if such differences can be measured among other experienced temporary health states.

Our findings suggest that the community-based values for avoiding health events related to pH1N1 illness and vaccination are consistent with the severity of the outcomes. These data also suggest that the public places a greater value on preventing outcomes in children, compared to adults, consistent with previous findings from seasonal influenza. The valuations derived from these data can be used along with other decision-making factors during the development of pandemic influenza vaccination programs in the U.S. and the allocation of future pandemic vaccine supplies.

## References

[1] National Center for Immunization and Respiratory Diseases (2009). Use of influenza A (H1N1) 2009 monovalent vaccine: recommendations of the Advisory Committee on Immunization Practices (ACIP), 2009.. MMWR Recomm Rep.

[2] Prosser LA, Bridges CB, Uyeki TM, Rego VH, Ray GT (2005). Values for preventing influenza-related morbidity and vaccine adverse events in children.. Health Qual Life Outcomes.

[3] Prosser LA, Payne K, Rusinak D, Shi P, Uyeki T (2011). Valuing health across the lifespan: health state preferences for seasonal influenza illnesses in patients of different ages.. Value Health.

[4] Johnston SS, Rousculp MD, Palmer LA, Chu BC, Mahadevia PJ (2010). Employees' willingness to pay to prevent influenza.. Am J Manag Care.

[5] Gold MR (1996). Cost-effectiveness in health and medicine.

[6] Dennis JM (2010). KnowledgePanel® Design Summary: KNOWLEDGEPANEL® OVERVIEW. Knowledge Networks.. http://www.knowledgenetworks.com/knpanel/docs/KnowledgePanel(R)-Design-Summary-Description.pdf.

[7] (2009). RESPONDENT INCENTIVES FOR KNOWLEDGEPANEL®.. http://www.knowledgenetworks.com/ganp/irbsupport/docs/KN%20IRB%20Doc%20-%20Section%204%20-%20Respondent%20Incentives.doc.

[8] Johannesson M (1996). Theory and methods of economic evaluation of health care.

[9] Efron B (1987). Better Bootstrap Confidence-Intervals.. Journal of the American Statistical Association.

[10] Attema AE, Brouwer WB (2010). The value of correcting values: influence and importance of correcting TTO scores for time preference.. Value Health.

[11] Hanley JA, Negassa A, Edwardes MD, Forrester JE (2003). Statistical analysis of correlated data using generalized estimating equations: an orientation.. Am J Epidemiol.

[12] Lin LI (1989). A concordance correlation coefficient to evaluate reproducibility.. Biometrics.

[13] Eisenberg D, Freed GL (2007). Reassessing how society prioritizes the health of young people.. Health Aff (Millwood).

[14] Eisenberg D, Freed GL, Davis MM, Singer D, Prosser LA (2011). Valuing health at different ages: evidence from a nationally representative survey in the US.. Appl Health Econ Health Policy.

[15] U.S. Department of Health and Human Services USDoHS, editor (2008). Guidance on Allocating and Targeting Pandemic Influenza Vaccine.. http://www.flu.gov/individualfamily/vaccination/allocationguidance.pdf.

[16] Wittenberg E, Halpern E, Divi N, Prosser LA, Araki SS (2006). The effect of age, race and gender on preference scores for hypothetical health states.. Qual Life Res.

[17] Gaskin DJ, Arbelaez JJ, Brown JR, Petras H, Wagner FA (2007). Examining racial and ethnic disparities in site of usual source of care.. J Natl Med Assoc.

[18] Lillie-Blanton M, Martinez RM, Salganicoff A (2001). Site of medical care: do racial and ethnic differences persist?. Yale J Health Policy Law Ethics.

[19] Weinstein MC, Torrance G, McGuire A (2009). QALYs: the basics.. Value Health.

[20] Brazier J, Rowen D, Tsuchiya A, Yang Y, Young TA (2011). The impact of adding an extra dimension to a preference-based measure.. Soc Sci Med.

[21] SteelFisher GK, Blendon RJ, Bekheit MM, Lubell K (2010). The public's response to the 2009 H1N1 influenza pandemic.. N Engl J Med.

[22] Prosser LA, Kuntz KM, Bar-Or A, Weinstein MC (2003). Patient and community preferences for treatments and health states in multiple sclerosis.. Mult Scler.

[23] Ubel PA, Loewenstein G, Jepson C (2003). Whose quality of life? A commentary exploring discrepancies between health state evaluations of patients and the general public.. Qual Life Res.

[24] Baron J, Asch DA, Fagerlin A, Jepson C, Loewenstein G (2003). Effect of assessment method on the discrepancy between judgments of health disorders people have and do not have: a web study.. Med Decis Making.

[25] van Hoek AJ, Underwood A, Jit M, Miller E, Edmunds WJ (2011). The Impact of Pandemic Influenza H1N1 on Health-Related Quality of Life: A Prospective Population-Based Study.. PLoS One.

